# An Undergraduate Interprofessional Experience with Self-Learning Methodology in Simulation Environment (MAES©): A Qualitative Study

**DOI:** 10.3390/nursrep12030043

**Published:** 2022-06-23

**Authors:** Giulio Fenzi, José Luis Díaz-Agea, David Pethick, Rocío Bertolín-Delgado, Noelia Hernández-Donoso, Luis Lorente-Corral

**Affiliations:** 1 Nursing Department, Universidad Católica San Antonio de Murcia, 30107 Murcia, Spain; gfenzi@ucam.edu; 2 Stroke Department, Royal Devon University Healthcare, Exeter EX2 5DW, UK; dave.pethick@nhs.net; 3 Nursing Department, Universidad Católica de Valencia, 46001 Valencia, Spain; rocio.bertolin@mail.ucv.es (R.B.-D.); noelia.hernandez1@mail.ucv.es (N.H.-D.); luis.lorente@ucv.es (L.L.-C.)

**Keywords:** simulation, interprofessional education, nursing education, medical education, crisis resource management, MAES©

## Abstract

This article describes the impact that a Self-learning Methodology in Simulated Environments can have on Interprofessional Education within a Crisis Resource Management simulated scenario. We used a qualitative approach. It is divided into three phases: study and design, plan of action, and analysis and evaluation. During the first phase of the study, there emerged a poor use of Interprofessional Education in the nursing and medical degrees, and it became apparent that there was a need for an implementation. Due to the possibility for better training for both technical and non-technical skills within Crisis Resource Management, a simulation scenario within this setting has been established as a learning baseline objective. The technique used to develop the scenario in the second phase of the study was the Self-learning Methodology in Simulated Environments. Its structure, comprising six items, was previously demonstrated in the literature as appropriate for healthcare degree students. The main result of the third phase shows an overall acceptance of an Interprofessional Education within Self-learning Methodology in Simulated Environments during the practice of a Crisis Resource Management scenario. The integrated application of a Self-learning Methodology in Simulated Environments, Interprofessional Education, and Crisis Resource Management result in a synergistic combination that allows students to share knowledge, technical, and non-technical skills using an innovative learning method.

## 1. Introduction

In healthcare, patients meet different professionals and specialists. Patients’ safety and satisfaction throughout all of their health-processes are the basis for patients’ care [1]. All healthcare professionals must link, train, and share their knowledge and competences to deliver a good standard of care that maintains patient safety [2]. The need for creating working links and sharing competences inside healthcare showcases the importance of training and improving knowledge, not only as a person or single professional, but also as an interprofessional team. When doctors and nurses train together, their learning is enriched and much more adjusted to reality than in traditional learning [3].

Interprofessional education (IPE) refers to “occasions when two or more professionals learn with, from and about each other to improve collaboration and the quality of care” [4,5]. IPE promotes interdisciplinary collaboration and teamwork [6], reduces the barriers and preconceptions prevailing among various healthcare groups, and promotes professional competencies [7,8]. IPE allows for the engagement of different health workers or students. These qualities evince IPE’s potential to be applied to both pre-graduate and post-graduate studies [9]. In 2016, the International Nursing Association for Clinical Simulation and Learning (INACSL)’s Standards of Best Practice: Simulation^SM^ published a standard of best practice specific to simulation enhanced interprofessional education (Sim-IPE) [10]. The standard is based on four criteria: (a) using a theoretical or conceptual framework, (b) using best practice in the design and development of Sim-IPE, (c) recognizing and addressing potential barriers, and (d) devising an appropriate evaluation plan. Despite a recently trending interest in interprofessional education, there remains a considerable gap in its study especially due to a lack of rigorous methods, design, implementation, and the difficulties of inserting a specific module of IPE inside the very stretched healthcare pre- and post-graduate programs [11].

One of the most effective methodologies that aids training for both technical and non-technical skills, which takes advantage of interprofessional education, is Crisis Resource Management (CRM). CRM is “*the ability to command and control all the resources at hand in order to execute care as planned and to respond to problems that arise*” and it includes a focus on communication, teamwork, decision making, the coordination of staff, leadership, and relations between team members [12]. Medical CRM is connected to Crew Resource Management, which was embraced by and formally created in aviation more than 20 years ago [13]. In a clinical practice, a small group of providers can work together, especially in stressful situations (e.g., cardiac arrest, difficult intubation, life-threatening situations inside theatres, etc.). We may meet different health professionals who join as a team to provide the best practice and care to a patient. In all these situations, medical and nursing staff are working not only alongside and between professionals, thus necessitating the implementation of IPE and CRM education, but also with pre-graduate medicine and nursing students. The potential application of IPE in pre-graduate settings can help future health professionals face more realistic situations during their training and become familiar with sharing and delegating the appropriate knowledge and skills, thereby creating a good team environment to safely care for patients [14,15,16].

To help health professionals in training, an experimental and reflective learning opportunity has been developed worldwide: the highly realistic clinical simulation [17]. A simulation can enable the training of both technical and non-technical skills (communication, leadership, coordination, prioritization, etc.), which makes it one of the best evaluated methods in the training of health professionals [10,18]. There exist various methodologies of clinical simulation. The main differences are found in the choice of the learning objectives. Simulation-based learning (SBL) is initially conducted by considering the objectives [19] which are set by the education center, by the course instructor, or by the training needs at an educational (university or course) or health (hospital) context. The simulation instructor is normally the one who manages and plans the session, sets the time, and ensures that the learning objectives are achieved [20].

An alternative to SBL is the Self-Learning Methodology in a Simulated Environment (Spanish acronym: MAES©) [21]. This innovative methodology, designed by Díaz, Leal, and García [22], and started in 2012 in the Catholic University of Murcia (Spain), is based on constructivist and situational learning [23]. MAES© is divided in six stages: pre-briefing, homework, briefing, simulation, debriefing, and exposition. It puts the student/learner at the center of their own learning throughout the whole process. During the pre-briefing, the learners are the ones who, guided by the facilitator, are in charge of choosing their learning needs, their learning objectives, and the competences that they want to learn or train. This can be done thanks to the group factor which helps to create a safety environment where learners can meet to share the strengths and weaknesses of their learning. Students are also in charge of designing the scenario (as homework). The group who designs the scenario will then give a quick handover (briefing) to a different group, following the SBAR method (Situation, Background, Assessment, and Recommendation) [24] The group that receives the handover enters the simulation room and performs a 10–12-min simulation. Once they are back in the debriefing room, the group who designed the scenario, with the help of the facilitator, guides the debriefing following the GAS method (Gather, Analise, and Summarize) [25] or another style of structured debriefing. The MAES© experience ends with the exposition phase. This phase is still part of the MAES© structured debriefing. The learners that provided the knowledge need to close the gap or eliminate the deficiencies detected in the first session. They can do so with different methods: presentations, online tests, practical training, sharing their experiences, or inviting someone who personally experienced the simulated scenario in real life [26]. A MAES© facilitator assumes a secondary but essential role in the process, helping the group-factor, guiding the learners, and assuring that they keep focused on the learning objective they set themselves [21].

Researchers have agreed about the importance of using healthcare simulation as a vehicle for the multidisciplinary and even interprofessional training of healthcare providers, especially in CRM training [27,28,29]. SBL is the simulation method predominantly used during simulation training and courses although diverse studies have shown that the MAES© method is well accepted by the students and facilitators both in university and clinical settings [30,31,32,33]. No studies have been found applying MAES© in an interprofessional simulation yet.

The objectives of this study were to determine the need for interprofessional training in pre-graduate nursing and medicine students, to implement an IPE experience applied to CRM simulation training using MAES©, and to evaluate participants’ perceptions of the MAES© method applied in IPE CRM training.

## 2. Materials and Methods

### 2.1. Settings

The Catholic University of Valencia (Spain, CUV) uses a building called Virtual Hospital (VH) for clinical simulation to train their students. At this location, students and professionals can use a very modern structure which mimics some hospital environments. VHs a have triage, a resus room, an emergency department observation area, general hospital wards, intensive care rooms, theatres, an obstetric room, and several other specialty rooms alongside laboratories and debriefing rooms. Although future nurses and doctors spend a lot of time in the same building, they do not share activities, classes, or simulations.

### 2.2. Design

We planned a qualitative study with formative action [34]. We were first interested in finding out what educational needs the simulation center had regarding interprofessional education, and then implementing a pilot joint training approach programmed with MAES© method as a tool and evaluating its impact. Qualitative study with a formative action is commonly utilized in education and social science contexts [35]. It consists of studying and analyzing a situation, with the intention of proposing a change to improve it, using a formative action. The researcher also becomes an agent of the change [36]. This study can be divided into three phases: detection of training needs (1º phase-Research), explication, preparation and application of the intervention with simulation MAES© (2º phase-Action), and evaluation and collection of evidence related to the results (3º phase-analysis and evaluation).

### 2.3. Participants

The study was conducted in the Catholic University of Valencia between January and March 2022.

To obtain information on the university objectives and learning needs, we considered the opinion of the expert in clinical simulation at CUV. He is a male nurse and professor, head of Clinical Simulation Module for nursing degrees, and is part of the organization of the Clinical Simulation Module for medicine degrees. His position made him our key informant. He was able to give us a general overview of the clinical simulation in his university. In addition, he served as the link between medicine and nursing by conducting a personal interview (1º phase). In this phase, we also created a group of students who decided to apply to the study, investigate their learning needs regarding CRM using a brainstorming technique, and apply the MAES© method. Recruitment was done for convenience, not using probability criteria. We finally chose those students who met the inclusion/exclusion criteria (Table 1). The same group of students that were chosen in the first phase participated in phases two and three. The participants were a total of 13 students (6 nursing and 7 medical students). Of the 13 participants, there were 12 women and 1 man, with an average age of 24.5 (SD 5.09). The group was led throughout the study by a researcher with MAES©, IPE, and clinical simulation experience.

### 2.4. Procedure

#### 2.4.1. First Phase

The interview was designed by the research team before it was performed. The content of the questions was agreed upon by the team before the final draft was accepted. The research team validated the poll questions after several meetings (for content validity). The interview was completed online in January 2022. A male researcher (GF) who had experience in the management of simulations and IPE conducted the interview with open questions (Table A1) so that the interviewed expert could tell his opinion in a semi-structured way. The result of the interview was used to identify and analyze the need for interprofessional simulation in the university and to choose an appropriate scenario/situation which could help interprofessional education.

In phase 1, the researcher also performed a “brainstorming session” with the group of students according to the MAES© pre-briefing standards. The technique helped the group to set their learning needs and their learning objectives. The participants analyzed their existing knowledge about CRM and shared what they already knew and decided what they wanted to learn.

#### 2.4.2. Second Phase

The first session of MAES© took place on 4 February 2022.The researcher (GF), an expert and facilitator of MAES© methodology, explained how it works and its characteristics to the students. The session lasted 240 min. MAES© standards were applied throughout the session; operational work groups were established, the psychological safety of the participants was worked on through group dynamics, and the simulation norms and the MAES© structure were explained. To establish the workgroups, the researcher divided the nursing students and the medical students. Asking questions about their interests (hobbies, pets, music, family members, birthday, travels, etc.) he managed to create 6 groups: 5 couples (1 nurse and 1 doctor) and 1 trio (1 nurse 2 doctors). Once the work teams were settled, the whole group proceeded to decide how to divide the work. This division left the 13 students divided in 3 definitive workgroups: one couple designed the scenario, the trio performed the simulation, and the remaining group were observers.

The facilitator explained how to design an MAES© case to the participants, providing them with resources for this (a template for the design and management of scientific evidence). The team who designed the scenario provided evidence about the learning objectives that were decided upon by the entire group. A month was given for this homework. During this time, the facilitator was available to resolve doubts or provide support for the design of the scenarios and the search for scientific evidence.

On 1 March, the second session of MAES© took place. This time, the team who designed the scenario helped to prepare the simulation room so that the team who had to perform the simulation could do so, while the observers remained in the debriefing room. The debriefing and the exposition also took place on the same day.

Both sessions took place at the VH. The first session was performed in a debriefing room. The second took place in two different rooms (simulation room and debriefing room). The debriefing room allowed all the participants to watch what was happening while the simulation was performed in the ITU simulation room. For the simulation, the group used a manikin, resuscitation, and airway equipment. The simulation session lasted 1 h (5-min briefing, 10 min simulation, 30 min debriefing, and 15 min exposition phase).

#### 2.4.3. Third Phase

After their participation in the learning program with the MAES© method, the participants were asked for their perceptions on the experience through a focus group technique. The focus group lasted one hour and consisted of the students and the university expert. Information was collected about how they had felt during the sessions, aspects about collaborative work, their opinion about the CRM scenario that they worked on, the simulation experience, the competences shown, the interprofessional education experience, and their overall idea of MAES©.

### 2.5. Data Analysis

The audio/video files (online interview and focus group were recorded) were transcribed into a single document which, after careful and independent reading by two different members of the research team with broad experience in qualitative research (GF, JLDA), served as the basis for categorization and content analysis [37]. The qualitative analysis software MaxQDA^®^ v18 (VERBI computer software, Berlin, Germany) [38] was utilized for the coding of the results. The units of significance that emerged from the independent analysis of the text were identified and coded, and then these were grouped into categories and subcategories, as a function of their similarity. Likewise, for the interpretation of data, the contrasted method by Colaizzi was utilized, as it is recommended for social research [39,40].

At all times, the researchers sought to maintain a reflective attitude to minimize the impact of their subjectivity on the process of data collection and analysis. To present this research, the transparency and quality guidelines proposed by the COREQ (consolidated criteria for reporting qualitative research) standards [41] checklist were followed (Table A2).

## 3. Results

### 3.1. Study and Design: Investigate and Set Learning Needs

#### 3.1.1. Expert Interview

The analysis of the interview with the university expert (his characteristics can be found in Section 2.3.) provided the following data that were organized into four categories: nursing and medical university training relationships, interprofessional education, MAES©, and CRM. In the interview, it emerged that nursing and medicine are two professions with many common objectives. Even with a visible and recognizable common ground during their working lives, the students’ interactions during their university education are very limited in time and space.

*“They are future professionals that will share many objectives and a great deal of knowledge: patients’ care, patients’ safety, health education, prevention etc. (…).” (I)*

*“Thinking about how long nurses and doctors live together after their graduation, in their work sites, I cannot believe how poor their relationship is during university. They barely see each other (…).” (I)*

The interviewee analyzed the existing interprofessional education related to his personal and university experience. The focus was pointed towards the common educational objectives.

*“* *Universities focus on developing nurses and doctor students’ critical thinking, their ability to face difficult situations, and improving their patients’ care (…).” (I)*

The IPE’s existing training in the CUV was analyzed. It emerged that there were IPE experiences, but that they were punctual and focused on delivering specific training to one of the two groups of students. The experiences were based on solving clinical cases by mixed groups. This type of situation does not completely exploit the competences and learning abilities of both categories of students.

*“If we clearly know what our common educational objective is, something is wrong when we use IPE only for some very punctual and specific training (…).” (I)*

*“We use IPE training to develop and train only specific techniques in a more theoretical way. This does not allow us to develop and train non-technical skills. They work together in the same room, but they do not solve the case together (…).” (I)*

The university expert’s desire to improve IPE was visible. During the interview, we could see that there was a will to assure that all the learning possibilities (planned and not planned) created by joining students from two different degrees were exploited. The interviewee determined that the best method towards this end was to repeat a clinical simulation. This method helps to deliver competences and train both technical and non-technical skills.

*“We observed the medical students use nursing students only to realise that technique alone cannot solve the problem, while nursing students referred to doctors only to ask prescriptions. This does not allow us to develop and train non-technical skills, whereas clinical simulation is more appropriate and could help students to experience more realistic scenarios (…).” (I)*

*“The students need to live experiences; they cannot base their training only on theory. Simulation can definitely help professors, tutors, and facilitators to deliver knowledge (…).” (I)*

The interviewee, who knew SBL and MAES© methods, focused his attention on the second, analysing its potential for IPE training. He mainly focused on the self-learning characteristic of the MAES© method and its collaborative learning nature.

*“With SBL, students solve a case given by an expert and the relationships between nurses, and medical students may be limited to the simulation and the learning objectives decided by the facilitator. With MAES©, the experience could be amplified as the students not only solve the scenario, but also decide what they want to learn and then design it (…).” (I)*

*“With MAES simulation, the medical students focus on their competences and the nurses on theirs, but for the first session, the homework and the structured debriefing could guarantee shared opportunities and the peer work could break barriers that students from different degrees usually have (…).” (I)*

During the interview, and in accordance with the university learning objective, the expert individuated a theme that was considered appropriate to performing IPE simulation training: CRM.

*“How students can manage a crisis could be the best theme to work on (…).” (I)*

*“In my opinion, CRM helps to share knowledge but it does not focus on how well you perform techniques or on who is the best in the team. It lets us focus on how students face a crisis together, as a team, something that we have less of an opportunity to train for in separate nursing and medical universities (…).” (I)*

#### 3.1.2. Students’ Learning Needs and Baseline Learning Objectives

As a consequence of the interview with the university expert, the group agreed to choose CRM (Crisis Resource Management) as the theme of the simulation. Using a brainstorming technique, the group decided what they wanted to learn and shared what they already knew, as shown in Table 2.

### 3.2. Action: IPE Planning and Simulating with MAES©

As for the results of the first MAES© session, the researcher helped the participants to divide themselves into six teams, who then decided to act as three groups:

- Group 1: design scenario, look for information to answer baseline learning needs during the exposition phase in the debriefing (one student nurse, one student doctor)
- Group 2: perform simulation and participate in debriefing (one student nurse, two student doctors)
- Group 3: observe and participate to the debriefing (four student nurses, four student doctors)

The group that designed the simulation did so in accordance with the template standardized for MAES© (the designed scenario is in Table A3). The case was set in a pre-Surgical area. The patient was a young female with acute abdominal pain that required emergency surgery. She needed airway management, and it was during her airway isolation that a crisis situation arose (CRM situation). To exacerbate the crisis, the group who prepared the simulation decided to give different roles and characteristics to each member of the group who was performing the simulation. The members of the simulating group did not know the role of their colleagues. In doing so, they aimed to create a stressful situation and reproduce a scenario where it was necessary to use CRM techniques. The roles were:

- Doctor 1: Emergency doctor who needed to manage the airway, even if she was not an expert. An authoritarian leader unopen to accepting others’ ideas and possessing a short temper.
- Doctor 2: Anesthetic doctor, expert in managing (obstructed?) airways. She could not give suggestions because she wanted to be the center of the situation and she had to try to take over the airway management from the ED doctor.
- Nurse: She needed to react to what she saw as she would have done in a real situation.

At the end of the simulation, the standardized debriefing following the MAES© standards took place and was guided by a facilitator. The students analyzed their strengths and weaknesses and implemented their knowledge (exposition phase) [28,29]. During the exposition phase, the group who designed the scenario answered the questions and emerged during session 1 and listed in Table 3. They did so with a PowerPoint^®^ presentation.

### 3.3. Evaluation: Perception of IPE with MAES© and CRM Experience Outcome

The activity that used the MAES© CRM simulation and IPE was analysed with a focus group (Table A4) at the end of the simulation learning session. After the literal transcription of the focus group, three categories and nine subcategories were found (Table 3).

#### 3.3.1. Category 1: MAES

The participants identified many positive aspects. One of the most frequently mentioned was the self-learning method and its link with the intrinsic motivation.

*“I liked the fact that I had to decide for myself about what I had to learn based on what I want, my interests, and how I wanted to spend my knowledge on my work. In other words, we were moved by our intrinsic motivation, and it made us more enthusiastic (…).” (GF3)*

*“Knowing our student-colleagues were the ones who were performing the simulation motivated us even more to design a simulation case that had the best realistic elements possible (…).” (GF1)*

Students also noticed that MAES© allowed them to keep lessons more dynamic compared to normal classes. They noted that there was more dynamism compared with their previous experience with SBL.

*“I think MAES is more practical and dynamic than a normal classroom. I found it even more dynamic than the clinical simulation I used to do (…).” (GF6)*

*“This MAES experience induced us to work, investigate, share, and learn using a very dynamic approach that made our jobs less heavy than a normal class (…).” (GF2, GF3, GF11)*

Finally, the structured debriefing with the exposition phase was seen as a great opportunity to complete participants’ learning and share their experiences.

*“Analyse together, during the debriefing, what went well and what could have better helped us to focus and better understand the scenario and the subject. The exposition phase, at the end of the debriefing, also helped to better fix the ideas and answer our own questions. It was the best way to close the learning circle (…).” (GF1, GF12)*

The students agreed that the homework was something that could cause additional stress on an already busy University table, although they admitted that they overcame this problem thanks to the motivation that was generated by working in groups.

*“I think the problem is homework and how at the beginning it is difficult to fit it into our already busy timetable. Although, I have to say, the group-work is motivating and the perception that we were really working for our own knowledge helped me to find an alternative solution for organisation (…).” (GF4)*

#### 3.3.2. Category 2: IPE

Speaking of interprofessional education, participants firstly analysed the actual situation of IPE at their university. They agreed about the need to practice with IPE, but that its emergence in their experience was rare during their university careers, and they thought that the timetable could be used differently.

*“In the future we will work together, and it is important to have experience together in university, but these experiences are rare. I can count three or maybe four times during my time in university. Many times, we (nursing students) have simulation on the same day as medical students and even with the same subject. Nevertheless, the university does not place us together. I think we are missing opportunities (…).” (GF7, GF9)*

Secondly, the participants analysed the potential positive and negative aspects of IPE training. Although they agreed on the possibility of an initial difficulty when adapting to students from a different degree and with different education and careers, they thought that IPE could enable an opportunity for implementing teamwork and communication, with a better understanding of all the situations.

*“I think at the beginning, it is difficult, and we could be a bit shy or have preconceived notions towards our colleagues from a different career (…).” (GF1, GF2, GF4)*

*“I do not think the problems with IPE are many, and even if they are, I think the positive aspects are more important. IPE allows us to see and touch a different view and a different way of working, even if it is with the same objective: patient care. It also improves teamwork and communication (…).” (GF2, GF5, GF10)*

Their opinions after the IPE simulation with MAES demonstrated the positive effect it had on their perception of the simulation, the teamwork, and the increased respect towards their student-colleagues from a different degree.

*“Working alongside our future doctor colleagues helped us to put ourselves in their position and better understand their work and decisions. MAES and its self-learning method unified with its collaborative learning characteristic accentuated teamwork, helped communication, and raised our mutual respect (…).” (GF8)*

*“Working with MAES during IPE made me meet someone with similar interests (patients’ care and patients’ safety) but at the same time made my group face the work from two different points of view. This enriched my experience (…).” (GF1, GF2)*

*“MAES structured debriefing helped us to gain a better understanding of the situation from both points of view (nurse and doctor). It also helped to improve our empathy towards our colleagues (…).” (GF7)*

#### 3.3.3. Category 3: CRM

The university expert and the students chose Crisis Resource Management as the simulation scenario during the first phase of the study. The preparation of the case, the simulation, and the structured debriefing with the exposition phase helped them to gain a better understanding of this subject. They thought that it was useful for two different reasons. Firstly, they thought that CRM was a great link to real situations and helped to minimize risks.

*“With CRM we can see and train many non-technical skills that help us in the future to reduce risks for both the patients and the healthcare professionals (…).” (GF4)*

Secondly, the positive training opportunity that CRM gave them made them reflect on possible future job experiences, sharing competences between different health professionals.

*“CRM helped us to better understand decision-making and how we should face a crisis in our team, and I think it is very useful when thinking of my professional future (…).” (GF3)*

*“Communication, empathy, teamwork, leadership…they are all aspects that we can train and learn with CRM for our future jobs. It has been a great opportunity to do so with IPE since it was even more realistic (…).” (GF6)*

## 4. Discussion

Our study is the first to evaluate MAES© applied to Interprofessional Education with a simulation. The study investigated the IPE situation at CUV and examined good methods to improve it. The results of this study are the basis for further research on the best way students from different health professions (doctors and nurses), who will eventually have interdependent jobs, can collaboratively learn together

The main findings account for the positive impact that interprofessional learning can have on undergraduates, provided that the learning model is adequate. In this sense, the MAES© method has been successfully used for this purpose.

The first finding of this study is the lack of IPE training during the nurses and doctors’ university careers, although the literature marks the common ground of education between these two universities [42]. Both Students and university experts point out that IPE training at CUV is used to develop and train only specific techniques in a more theoretical way. During the investigation, we applied IPE and approached students’ learning needs with a more practical method: simulation [43]. Students need new learning methods that are more dynamic, interactive, and based on intrinsic motivation. IPE allowed them to see and experience different ways and views of working towards the same objective: patient care. It also improved teamwork and communication. During IPE, the students could work in a group using a cooperative learning method which helped their motivation. It also helped to improve empathy, communication, and teamwork. All those aspects comprise the basis of the hospital teamwork that healthcare students will find once they finish university.

The available literature supports the idea that IPE is fundamental to training teams and aspects such as teamwork, coordination, communication, and good problem-solving abilities. Most of these skills are fundamental non-technical skills for health professionals and they are difficult to train and teach at universities [3]. Crisis Resource Management is a situation that professionals experience daily. It is very important for healthcare, as any error or rupture in teamwork may affect patients’ safety and care [44]. It includes many aspects (teamwork, communication, and leadership) and allows for the development of both technical and non-technical skills. Students’ awareness that nurses and doctors will share many hours and spaces during their work career motivates them to be ready to work together. The students nowadays need to feel ready to work when they finish their university education, especially for those who are requested in healthcare fields. This affects their willingness to learn and to be receptive to new methods [45].

Although this study provides results from a small pool of participants, the main finding is the overall acceptance of IPE with simulation especially when applying the MAES© method. Based on cooperative learning, peer education, constructivist, and situational learning, MAES© simulation is a great method for application to IPE with simulation for its structure, practicality, and dynamicity [23]. For the duration of their work using MAES©, students must work together and move towards answering the learning needs they have set themselves. The group factor and teamwork are two very important aspects shared by IPE and MAES©. The fact students with different preparations and backgrounds are sharing knowledge and different points of view can help them to reach their true potential. While the simulation itself is lived similar in MAES© and SBL, there are three different moments when IPE can further benefit from MAES©.

As shown in the results of the study, the setting of learning needs (first session), homework, and debriefing can guarantee more sharing opportunities. In the first session, the peer education and the constructivist learning method helped to break the barriers existing between the nursing and medical students. The role of the facilitator is very important as the elimination of friction and the creation of an atmosphere of respect are necessary. The previously broken barrier lets future nurses and doctors combine their knowledge and skills during homework. Collaborative learning, self-learning, and intrinsic motivation help to complete it. During the debriefing we can see the real result of the work. It is in this moment when the whole group benefits from sharing experiences, knowledge, and answers to the questions they completed in the first session [31].

The main limitation of this work is the local and reduced nature of the study. As it is a single experience, it is difficult to generalize the results to other contexts. The external validity of the study could be improved by conducting more experiences in other university contexts (multicenter study), with more participants and using other methods in addition to qualitative research. However, we can consider this work as a pilot study that would lay the foundations for a broader interprofessional learning model based on the empowerment of students thanks to the self-directed and peer-to-peer learning provided by MAES©.

## 5. Conclusions

The present study identifies the necessity of Interprofessional training for nursing and medical students. It associates the MAES© method to IPE training with simulation and an overall acceptance. The application of CRM in a simulation scenario results in a great opportunity for students who can familiarize with stressful situations among different professionals. MAES©, IPE, and CRM appear to be a great combination that allows healthcare professionals to share knowledge, technical, and non-technical skills using a dynamic and new method. With this experience the students were moved towards the results by their intrinsic motivation. The participants recognized it as a very positive experience although they recognized that the MAES© structure required them to find more time and new resources. In the future, it may be interesting to develop similar studies with a larger pool and implement the number of experiences of MAES© with IPE.

### 5.1. Limitations

This study refers to a short experience with a small group of students (13 students). The size of the group and the lack of different universities participating in this experience may affect the results.

### 5.2. Implications for Educational Practice

The necessity of building good work relationships among healthcare professionals emerged from this research. This necessity is not only limited to the work environment (post-graduate), but also applies to pre-graduate settings. Considering these aspects, IPE training can become a resource inside the nursing and doctor students’ university program. It helps to initiate an early-contact between two professions that have daily strong relationships during their working-life. It is necessary to involve future health professionals who will work interdependently in the learning process. Such a practice will allow health professionals to avoid miscommunication and to clearly understand and determine the skills of each team member.

The method used for training (MAES©) was suitable as it was a method that was wildly accepted by the participants. MAES© could be used in interprofessional educational contexts. It facilitates teamwork and a psychologically safe learning atmosphere. Peer education and self-directed learning methods, both used with the MAES© technique, can help participants to motivate themselves to improve their own learning. Creating a safe environment also helps to break the barriers between different degrees.

This pilot study has shown that joint training in non-technical skills (such as CRM) between medical and nursing students is highly satisfactory for the participants. The study used CRM as a good tool to work around interprofessional work relationships, focusing on communications, leadership, delegation, etc.

The implications of this preliminary study for educational practice are promising. Although this study has shown a general acceptance of the method (IPE with MAES©) and theme (CRM), further studies are needed for a more powerful and definitive recommendation.

## Figures and Tables

**Table 1:** Inclusion/Exclusion criteria.

Inclusion	Exclusion
Last year nursing students (4º year) Last year medical students (6º year) Previous clinical simulation experience	Previous experience with interprofessional training

**Table 2:** Baseline learning needs.

What Do We Know?	What Do We Want to Learn?
CRM was born in aviation.	What is CRM?
Teamwork and communication are two of the most important concepts in CRM. The qualities that define a team leader.	Is there any existing protocol we can follow to apply CRM? Who are the people and professionals involved in CRM? What are the techniques and concepts related to CRM applied in healthcare? Who is more appropriate to be a team leader in healthcare?

**Table 3:** Categories and subcategories of Focus Group.

Category	Subcategory
MAES©	Self-learning & intrinsic motivation
	MAES© compared with previous experiences
	Debriefing
	Aspects to improve
IPE	Present situation
	Positive Aspects
	IPE & MAES©
CRM	Link with work situation
	Positive aspects to train CRM

## Data Availability

Not applicable.

## Appendix A

**Table A1:** Interview.

Interview to the University expert		
What is your name?		
What is your role in the university		
Have you got any clinical simulation experience?		
Have you got any Interprofessional education experience?		
Do you interact with both nursing and medical degrees?		
What do you think about the relation that exist between nursing and medical students during their years in university, on a formation point of view?		
What is the main lack you have noticed during nursing and medical students simulation, thinking of no-interprofessional experiences?		
How can we help both students to think and learn in a more cooperative way?		
Do you know MAES©?		
Do you think MAES© can help interprofessional training? Can you motivate your answer?		
What do you think could be an appropriate case scenario to work with interprofessional MAES©?		
**No. Item**	**Guide Questions/Description**	**Reported on Page**
**Domain 1:**		1
**Research team and reflexivity**		
Personal Characteristics		1
1. Inter viewer/facilitator	Which author/s conducted the interview or focus group?	
2. Credentials	What were the researcher’s credentials? E.g. PhD, MD	1
3. Occupation	What was their occupation at the time of the study?	1, 5
4. Gender	Was the researcher male or female?	5
5. Experience and training	What experience or training did the researcher have?	5
Relationship with participants		5
6. Relationship established	Was a relationship established prior to study commencement?	4
7. Participant knowledge of the interviewer	What did the participants know about the researcher? e.g., personal goals, reasons for doing the research	4, 5
8. Interviewer characteristics	What characteristics were reported about the inter viewer/facilitator? e.g., Bias, assumptions, reasons and interests in the research topic	4, 5
**Domain 2: study design**		3–6
Theoretical framework		1–3
9. Methodological orientation and Theory	What methodological orientation was stated to underpin the study? e.g., grounded theory, discourse analysis, ethnography, phenomenology, content analysis	1, 3
Participant selection		4
10. Sampling	How were participants selected? e.g., purposive, convenience, consecutive, snowball	4
11. Method of approach	How were participants approached? e.g., face-to-face, telephone, mail, email	3, 4
12. Sample size	How many participants were in the study?	4
13. Nonparticipation	How many people refused to participate or dropped out? Reasons?	N/A
Setting		
14. Setting of data collection	Where was the data collected? e.g., home, clinic, workplace	3
15. Presence of non-participants	Was anyone else present besides the participants and researchers?	N/A
16. Description of sample	What are the important characteristics of the sample? e.g., demographic data, date	4
Data collection	17. Interview guide Were questions, prompts, guides provided by the authors? Was it pilot tested?	13, 18, 19
18. Repeat interviews	Were repeat interviews carried out? If yes, how many?	N/A
19. Audio/visual recording	Did the research use audio or visual recording to collect the data?	6
20. Field notes	Were field notes made during and/or after the interview or focus group?	6
21. Duration	What was the duration of the inter views or focus group?	4
22. Data saturation	Was data saturation discussed?	6
23. Transcripts returned	Were transcripts returned to participants for comment and/or correction?	6
**Domain 3: analysis and findings**		
Data analysis		
24. Number of data coders	How many data coders coded the data?	N/A
25. Description of the coding tree	Did authors provide a description of the coding tree?	N/A
26. Derivation of themes	Were themes identified in advance or derived from the data?	6
27. Software	What software, if applicable, was used to manage the data?	6
28. Participant checking	Did participants provide feedback on the findings?	N/A
Reporting		
29. Quotations presented	Were participant quotations presented to illustrate the themes/findings? Was each quotation identified? e.g., participant number	6–11
30. Data and findings consistent	Was there consistency between the data presented and the findings?	6–11
31. Clarity of major themes	Were major themes clearly presented in the findings?	6–11
32. Clarity of minor themes	Is there a description of diverse cases or discussion of minor themes?	6–11

**Table A2:** COREQ.

**MAES simulation**	
**MAES clinical simulation title:** CRM	
**Team planning the simulation:** RN-TEAM	
**Information provided to the simulator group prior to the simulation**	
**Debriefing**	**Clinical history & situation**
**Learning goals & discussion points**	**Situation**
1. What is CRM?	25 y.o. female patient who attended Emergency Department with 3 days history abdominal pain (RLQ), nausea and temperature. No vomit, no diarrhoea. In ED they performed XR, Bloods and CT scan before deciding she needed urgent appendicectomy.
2. Existing protocols	
3. People involved?	
4. Who is teamleader?	
**Technical & non technical skills**	**Background**
	Allergic to paracetamol. Smoker.
1. Technique to solve a CRM (check list)	**Assessment**
	FC 126, TA 87/52, FR 33, Sats 95%, Temp 39º. Patient with vomit, strong abdominal pain (8/10), GCS 13, pale, diaphoretic.
	**Recommendation**
	Assessment and secure airway to transfer her to theatre.

**Table A3:** MAES© Scenario.

**Clinical happenings** (Evolution of the clinical situation during the simulation)		
Team enter the emergency room and the patient is semi-conscious. She knows about her situation, but she is starting to crash and vomiting. Theatre call, they are ready to receive the patient Doctor starts to induce hypnosis Start first attempt of intubation: failed If doctor tries a second attempt without changing technique, it will fail If doctor changes the technique, it will succeed but IOT will be on their right lung and the patient will not manage sats >87% During Third attempt of IOT, patient will have an airway oedema and they will need a difficult intubation kit In every moment, actors have to react to stress and crisis following their characteristics.		
**Interventions** (Activities and interventions that the simulator team is expected to perform)		
**Intervention 1: Conflict resolution**		
	YES	NO
Activity 1: Allow team members to express their feelings		
Activity 2: Use different communication technique (reflexion, active listening, open questions)		
Activity 3: Help members to identify problems and possible solutions		
Activity 4: Facilitate problems solution		
Activity 5: Help members to actively solve problems		
**Intervention 2: Intubation and airway management**		
	YES	NO
Activity 1: Wash hands		
Activity 2: Select correctly oropharyngeal/nasopharyngeal airway		
Activity 3: Correct patient’s position and preoxygenation		
Activity 4: Aspiration of patient’s airway		
Activity 5: Insert airway management tool and verify correct position		
**Resources**		
	Mannequin and Actors	
	Instruction for the actors: **Doctor 1:** Emergency doctor who needed to manage airway, even if she was not an expert. Authoritarian leader, not opened to accept others’ ideas. Easy to get angry **Doctor 2**: Anesthetic doctor, expert in managing difficult airway. She could not give suggestions because she wanted to be the center of the situation and she had to try to take over the airway management from the ED doctor. **Nurse**: She needed to react to what she saw as she would have done in a real situation	
	Materials	
	Resus trolley, airway trolley, difficult airway trolley, monitor, cannulation materials, medication for definitive airway management	
**SCIENTIFIC EVIDENCES**		
**Themes**	**Reference**	
1-2-3	https://www.google.com/search?client=safari&rls=en&q=Simulaci%C3%B3n+cl%C3%ADnica+y+segurdad+en+urgencia (accessed on 3 February 2022) https://litfl.com/crisis-resource-management-crm/s+y+emergencias%3A+Emergency+Crisis+Resource+Management+(E-CRM)&ie=UTF-8&oe=UTF-8 (accessed on 3 February 2022)	
4	https://eds.p.ebscohost.com/eds/pdfviewer/pdfviewer?vid=0&sid=9ebca4d3-2d7c-479f-9903-716870e29339%40redis (accessed on 3 February 2022)	
Check List	https://www.fcchi.org.ar/wp-content/uploads/2020/02/Checklist-IOT-UTIA-HIBAfeb2020.pdf?fbclid=IwAR1oR575sOaUzLnwgNCENF47niua6iQ1N7GFsmxa-uARd5qg4zEYM9wXU (accessed on 3 February 2022)	

**Table A4:** Focus Group.

Focus Group
What is your name?
Have you got any clinical simulation experience?
Have you got any Interprofessional education experience?
What do you think about teamwork?
What do you think about the relation that exist between nursing and medical students on a formation point of view?
What do you think about the possibility to work with a doctor/nurse teammate?
Did you know MAES©?
Do you think MAES© can help interprofessional training? Can you motivate your answer?
